# Supplementary material for: Evaluation of Feeding Beta-Hydroxy-Beta-Methylbutyrate (HMB) to Mouse Dams during Gestation on Birth Weight and Growth Variation of Offspring
Source: Animals (Basel). 2023 Oct 15;13(20):3227. doi: 10.3390/ani13203227 (PMC10603694; doi:10.3390/ani13203227)
Supplement: Supplementary file 1 [file animals-13-03227-s001.zip › animals-2579667-supplementary.pdf]

Supplementary Materials

Table S1. Calculated nutrient composition of experimental diets, as fed.

	Diet[1]		
Nutrient	CON	LL	HL
Dry matter, %	87.87	87.50	84.21
Net energy, kcal/kg	2,492.30	2,481.60	2,385.10
Crude protein, %	18.00	17.92	17.23
Crude fat, %	5.00	4.98	4.89
Amino acids, total:			
Lys, %	1.05	1.04	1.00
Thr, %	0.69	0.68	0.66
Met + Cys, %	0.57	0.56	0.54
Trp, %	0.24	0.24	0.23
Ile, %	0.81	0.80	0.77
Val, %	0.90	0.90	0.87
Arg, %	1.15	1.14	1.10
His, %	0.48	0.48	0.46
Leu, %	1.59	1.59	1.53
Phe + Tyr, %	1.16	1.15	1.10
Minerals:			
Calcium, total %	0.76	0.82	1.42
Phosphorus, total %	0.54	0.54	0.52
Sodium, %	0.12	0.12	0.12
Chlorine, %	0.19	0.19	0.18
Magnesium, %	0.18	0.18	0.18
Potassium, %	1.17	1.16	1.12
Copper, mg/kg	12.0	12.0	12.0
Iron, mg/kg	101.0	100.0	97.0
Manganese, mg/kg	25.0	25.0	24.0
Zinc, mg/kg	57.0	57.0	54.0
Vitamins:			
A, IU/kg	5,079.0	5,076.0	4,874.0
D, IU/kg	1,001.0	1,001.0	961.0
E, IU/kg	89.0	89.0	86.0
K, mg/kg	0.75	0.75	0.72
Riboflavin, mg/kg	7.46	7.45	7.15
Niacin, mg/kg	49.71	49.62	47.66
Pantothenic acid, mg/kg	16.00	15.95	15.73
Choline, mg/kg	2,530.05	2,523.46	2,414.32
Biotin, mg/kg	0.34	0.34	0.33
B-12, mg/kg	0.02	0.02	0.02
Folic acid, mg/kg	2.28	2.28	2.28
Pyridoxine, mg/kg	6.99	6.99	6.99
Thiamin, mg/kg	8.14	8.13	8.14
HMB[2], mg/g	0.00	3.50	35.00
Analyzed nutrients:			
HMB[2], mg/g	0.00	6.12	36.80

[1]CON = Control; LL = Low level HMB supplementation; HL = High level HMB supplementation.

[2]β-Hydroxy-β-methylbutyrate

Table S2. Comparison of experimental control diet to nutrient requirements of mice (as fed).

Nutrient	CON	NRC[1]
Dry matter, %	87.87	-
Net energy, kcal/kg	2,492.30	-
Crude protein, %	18.00	18.00
Crude fat, %	5.00	5.00
Amino acids, total:		
Lys, %	1.05	0.40
Thr, %	0.69	0.40
Met + Cys, %	0.57	0.50
Trp, %	0.24	0.10
Ile, %	0.81	0.40
Val, %	0.90	0.50
Arg, %	1.15	0.30
His, %	0.48	0.20
Leu, %	1.59	0.70
Phe + Tyr, %	1.16	0.76
Minerals:		
Calcium, total %	0.76	0.50
Phosphorus, total %	0.54	0.30
Sodium, %	0.12	0.05
Chlorine, %	0.19	0.05
Magnesium, %	0.18	0.05
Potassium, %	1.17	0.20
Copper, mg/kg	12.0	6.0
Iron, mg/kg	101.0	35.0
Manganese, mg/kg	25.0	10.0
Zinc, mg/kg	57.0	30.0
Vitamins:		
A, IU/kg	5,079.0	2,398.0
D, IU/kg	1,001.0	998.0
E, IU/kg	89.0	31.0
K, mg/kg	0.75	0.99
Riboflavin, mg/kg	7.46	7.05
Niacin, mg/kg	49.71	14.99
Pantothenic acid, mg/kg	16.00	15.87
Choline, mg/kg	2,530.05	1,999.59
Biotin, mg/kg	0.34	0.20
B-12, mg/kg	0.02	0.009
Folic acid, mg/kg	2.28	0.51
Pyridoxine, mg/kg	6.99	7.94
Thiamin, mg/kg	8.14	5.07

[1]Nutrient Requirements of Laboratory Animals: Fourth Revised Edition, 1995. Washington (DC): National Academies Press (US); 1995. 3, Nutrient Requirements of the Mouse.

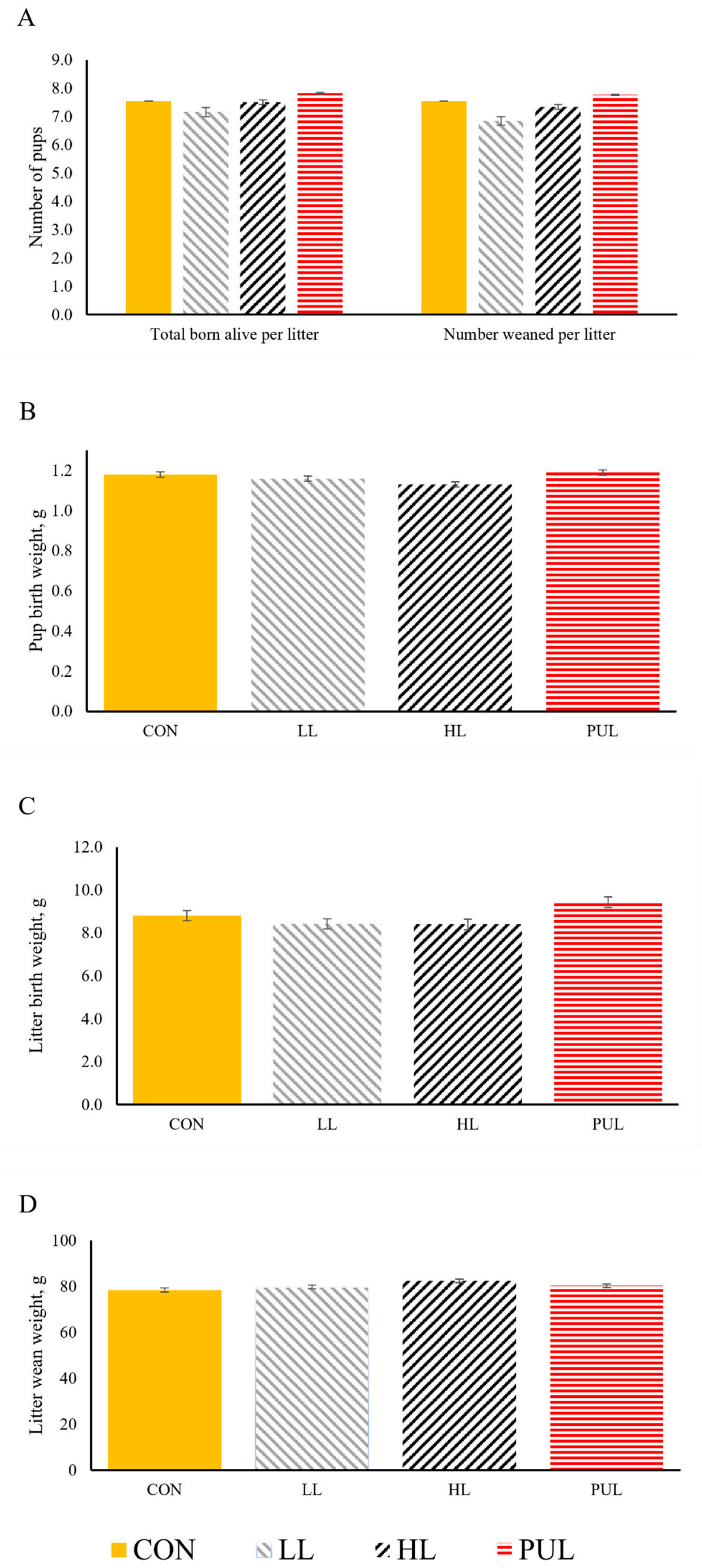

A
9.0
8.0
7.0
6.0
5.0
4.0
3.0
2.0
1.0
0.0
Number of pups
Total born alive per litter
Number weaned per litter
B
1.2
1.0
0.8
0.6
0.4
0.2
0.0
Pup birth weight, g
CON
LL
HL
PUL
C
12.0
10.0
8.0
6.0
4.0
2.0
0.0
Litter birth weight, g
CON
LL
HL
PUL
D
100
80
60
40
20
0
Litter wean weight, g
CON
LL
HL
PUL
CON
LL
HL
PUL

Figure S1. Effect of dietary treatment on pre-weaning performance of litters. (a) number born alive (SE = 0.51) and number weaned (SE = 0.55) for litters; (b) average pup birth weight for each treatment (SE = 0.07); (c) average litter weight at birth for each treatment (SE = 0.59); (d) average litter weight at weaning for each treatment (SE = 6.77). CON = Control; LL = Low level HMB supplementation; HL = High level HMB supplementation; PUL = Pulse dose of low level HMB during days 6 to 10 of gestation.

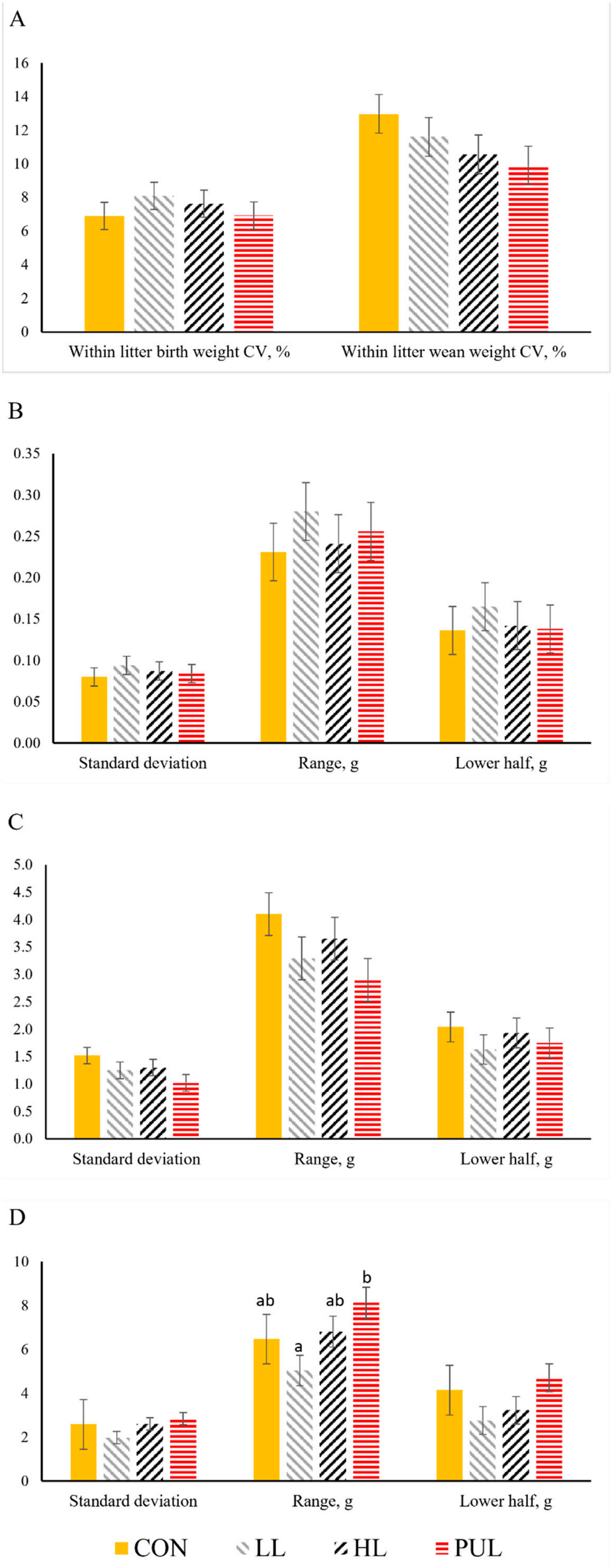

A
16
14
12
10
8
6
4
2
0
Within litter birth weight CV, %
Within litter wean weight CV, %
B
0.35
0.30
0.25
0.20
0.15
0.10
0.05
0.00
Standard deviation
Range, g
Lower half, g
C
5.0
4.5
4.0
3.5
3.0
2.5
2.0
1.5
1.0
0.5
0.0
Standard deviation
Range, g
Lower half, g
D
10
8
6
4
2
0
ab
a
ab
b
Standard deviation
Range, g
Lower half, g
CON
LL
HL
PUL

Figure S2. Effects of dam's dietary treatment on measures of variation in each litter. Standard deviation is defined as the standard deviation of individual pup weight within litter. Range is defined as the difference between the heaviest and lightest pup of each litter. Lower half is defined as the difference between the median and the lightest pup of each litter. (a) The within litter coefficient of variation (CV) for pup body weight at birth and weaning; (b) the standard deviation, range, and lower half values for pup body weights at birth; (c) the standard deviation, range, and lower half values for pup body weights at weaning; (d) the standard deviation, range, and lower half values for pup body weights at 8 weeks of age. CON = Control; LL = Low level HMB supplementation; HL = High level HMB supplementation; PUL = Pulse dose of low level HMB during days 6 to 10 of gestation.

[ab] Bars with different superscripts are different ($P < 0.05$) within each response variable.

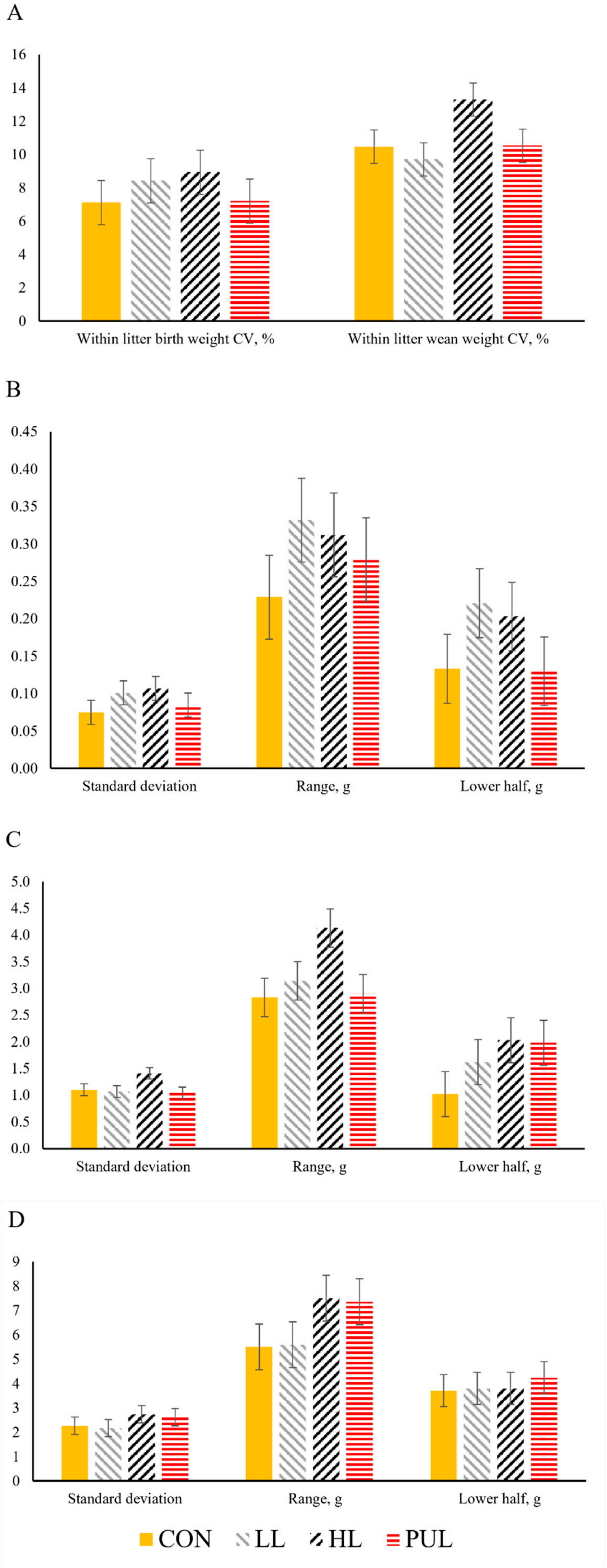
A
16
14
12
10
8
6
4
2
0
Within litter birth weight CV, %
Within litter wean weight CV, %
B
0.45
0.40
0.35
0.30
0.25
0.20
0.15
0.10
0.05
0.00
Standard deviation
Range, g
Lower half, g
C
5.0
4.5
4.0
3.5
3.0
2.5
2.0
1.5
1.0
0.5
0.0
Standard deviation
Range, g
Lower half, g
D
9
8
7
6
5
4
3
2
1
0
Standard deviation
Range, g
Lower half, g
CON
LL
HL
PUL

Figure S3. Effects of dam dietary treatment on measures of variation in litters with a minimum of 8 pups. The numbers of litters evaluated at birth were: CON = 8; LL = 5; HL = 6; PUL = 8. The numbers of litters for variation at weaning and 8 weeks were: CON = 2; LL = 3; HL = 5; PUL = 4. Standard deviation is defined as the standard deviation of weight within litter. Range is defined as the difference between the heaviest and lightest pup of each litter. Lower half is defined as the difference between the median and the lightest pup of each litter. (a) The within litter weight coefficient (CV) of variation at birth and weaning; (b) the standard deviation, range, and lower half values for pup weights at birth; (c) The standard deviation, range, and lower half values for pup weights at weaning; (d) the standard deviation, range, and lower half values for pup weights at 8 weeks of age. CON = Control; LL = Low level HMB supplementation; HL = High level HMB supplementation; PUL = Pulse dose of low level HMB during days 6 to 10 of gestation.

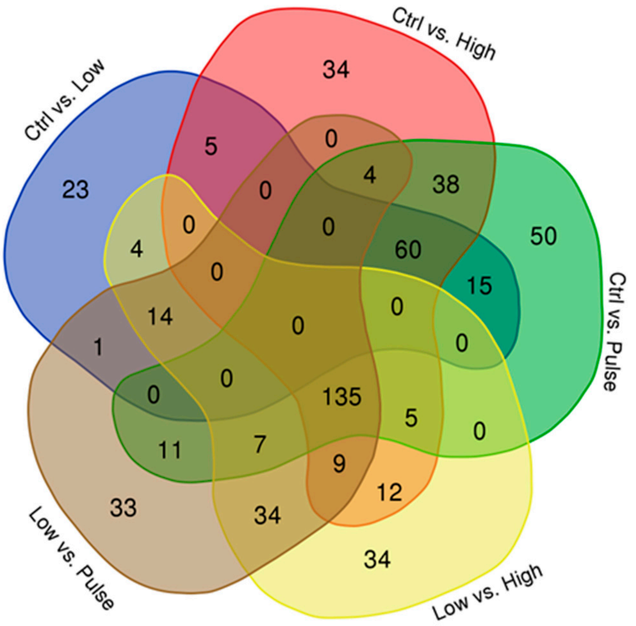

Figure S4. New Data Gene Analysis, 5-way Venn diagram.

Table S3. Gene ontology (GO): biological process assignments to genes differentially expressed between control and high level and pulse dietary HMB dosage groups.

ID	Name	Bonferroni	Genes from input	Genes in annotation
GO:0006811	Ion transport	4.69E-07	64	1956
GO:0017144	Drug metabolic process	3.48E-06	35	760
GO:0055085	Transmembrane transport	5.35E-06	59	1830
GO:0098660	Inorganic ion transmembrane transport	3.99E-05	41	1088
GO:0034220	Ion transmembrane transport	4.52E-04	47	1469
GO:0006812	Cation transport	4.69E-04	46	1423
GO:0098662	Inorganic cation transmembrane transport	2.66E-03	35	993
GO:0098655	Cation transmembrane transport	2.20E-02	36	1138
GO:0015672	Monovalent inorganic cation transport	1.36E-04	31	714
GO:0030029	Actin filament-based process	3.06E-02	29	829

Table S4. ToppGene Pathway analysis.

ID	Name	Source	Bonferroni	Genes from Input	Genes in Annotation
1269869	Striated Muscle Contraction	BioSystems: REACTOME	3.77E-06	9	34
82957	Arginine and proline metabolism	BioSystems: KEGG	1.95E-03	8	50
M2551	Arginine and proline metabolism	MSigDB C2 BIOCARTA (v7.1)	3.57E-03	8	54
M16024	Alzheimer's disease	MSigDB C2 BIOCARTA (v7.1)	4.45E-03	13	165
1269915	Type II Na+/Pi cotransporters	BioSystems: REACTOME	6.51E-03	3	3
83097	Alzheimer's disease	BioSystems: KEGG	6.61E-03	13	171
172847	Protein digestion and absorption	BioSystems: KEGG	2.31E-02	9	90
1457776	Miscellaneous digestion events	BioSystems: REACTOME	2.53E-02	3	4
212237	Mineral absorption	BioSystems: KEGG	2.61E-02	7	51
PW:0000034	Oxidative phosphorylation	Pathway Ontology	2.98E-02	7	52
1269868	Muscle contraction	BioSystems: REACTOME	4.43E-02	13	204
MAP00330	MAP00330 Arginine and proline metabolism	GenMAPP	4.67E-02	6	38

Table S5. Comparison of reproductive traits in mice and pigs.

Trait	Mouse dam	Sow
Age of sexual maturity	42-56 days of age	160-220 days of age
Length of gestation	19-21 days	113-115 days
Length of lactation, typical	21-28 days	21 days
Litter size	4-12	12-14
Within-litter birth weight CV, %	10%[1]	19%[2]
Placental type	Discoid[3]	Diffuse[4]
Nutrient exchange mechanism	Counter-current system[5]	Cross-current system[6]
Number of teats	10 (5 pairs)	12-16 (6-8 pairs)

[1]Value is inherent variation only, genetically identical mice housed in environmentally controlled rooms.

[2]Value includes genetic, environmental, and inherent variability.

[3]A placenta where chorionic villi are arranged in a circular plate.

[4]A placenta made up of villi diffusely scattered over almost the entire surface of the chorion.

[5]The maternal and fetal blood flows in opposite directions with vessels parallel to each other and is the most efficient nutrient exchange system.

[6]The maternal and fetal blood vessels cross each other creating one point of contact for nutrient exchange.
